# Accessing Anonymised Data from Ireland’s Maternal and Newborn Clinical Management System for Research Purposes

**DOI:** 10.12688/hrbopenres.13898.1

**Published:** 2024-06-04

**Authors:** Gillian M. Maher, Ali S. Khashan, Joye McKernan, Fergus P. McCarthy, Richard A. Greene

**Affiliations:** 1 INFANT Research Centre, University College Cork, Cork, T12YE02, Ireland; 2 School of Public Health, University College Cork, Cork, T12XF62, Ireland; 3 National Perinatal Epidemiology Centre, University College Cork, Cork, T12YE02, Ireland; 4 Department of Obstetrics and Gynaecology, University College Cork, Cork, T12YE02, Ireland

**Keywords:** Maternal and Newborn Clinical Management System (MN-CMS); electronic health record, clinical data; maternal and child health

## Abstract

**Background:**

Since 2016, maternity units across Ireland have been switching from paper clinical notes to an electronic health record called the Maternal and Newborn Clinical Management System (MN-CMS). Currently, four units have implemented the MN-CMS: Cork University Maternity Hospital (CUMH), University Hospital Kerry (UHK), Rotunda Hospital and National Maternity Hospital (NMH). The MN-CMS provides opportunity for new data-driven discovery to answer important research questions on maternal and child health.

**Aim:**

Provide detailed information on how a request can be made to access anonymised MN-CMS data for research purposes, as well as current timelines involved from initial request to data access.

**Methods:**

A request to collaborate should be made to the clinical lead within each maternity unit where data is being requested in the first instance. A detailed protocol and data collection sheet should be drafted and forwarded to the National Information Governance Group for approval. A Data Protection Impact Assessment should be completed, and permission to access data from each maternity unit must be applied for separately from each unit’s local Ethics Committee. Upon receipt of ethical approval, an application to the Local Information Governance Group must be submitted if requesting data from CUMH and/or UHK. Data can then be extracted and anonymised by the data manager at the respective unit and transferred securely to relevant project personnel.

**Results:**

The timeline from initial request to data access can range from approximately 6-12 months, depending on number of maternity units from which data is being requested and number of variables being requested from each unit.

**Conclusions:**

Accessing MN-CMS data for research can be a complex process. A national standardised process for managing the data is needed. This would allow a clear pathway to be developed for accessing data to facilitate new data-driven discovery in the area of maternal and child health.

## Introduction

Ireland’s Health Identifiers Act 2014 saw the introduction of Individual Health Identifier (IHI) numbers to uniquely identify individuals engaging with health services. It was successfully implemented during the COVID-19 pandemic to facilitate the vaccine schedule. The IHIs continue to be rolled out across Ireland to enable the linking of medical records held in different healthcare organisations ^1^.

Consequent to the introduction of the IHI, the Maternal and Newborn Clinical Management System (MN-CMS) was introduced in Ireland in 2016 ^2^. The MN-CMS project was designed in consultation with each of the 19 maternity units in Ireland to address the need for a Maternal and Newborn Electronic Health Record. With this system, pregnancy data in maternity services in Ireland were switched from paper clinical notes to an electronic health record, allowing all maternal and newborn information to be stored on one record. The data are collected during routine delivery of maternity care, including but not limited to, demographics, lifestyle factors, medical history, pregnancy factors including any complications of pregnancy, delivery data such mode of delivery as well as data relating to the newborn such as birthweight and gestational age. To date, four units, which together account for almost half the number of total births in Ireland, have implemented the MN-CMS: Cork University Maternity Hospital (CUMH), University Hospital Kerry (UHK), Rotunda Hospital and National Maternity Hospital (NMH).

The MN-CMS is an effective and efficient method of recording pregnancy and neonatal data and has the potential to improve patient care through better communication between health professionals and improved clinical audit capabilities. Furthermore, the MN-CMS provides opportunity for new data-driven discovery to answer a broad range of research questions on maternal and child health ^3^. For example, using anonymised MN-CMS data, we previously developed and validated prediction models examining a combination of risk factors to predict postpartum haemorrhage (PPH) and third- and fourth-degree perineal tears in a general obstetric population ^4, 5^. Additionally, other studies have used MN-CMS data to assess risk factors for antenatal pyelonephritis ^6^, to examine the impact of an electronic health record on task time distribution in a Neonatal Intensive Care Unit ^7^ and to examine institutional rates of unsuccessful operative vaginal delivery in a tertiary level maternity hospital in Ireland ^8^.

There is a general public consensus on the importance of using health information for purposes beyond direct patient care, with the COVID-19 pandemic further highlighting the need for sharing and coordination of data in Ireland ^9^. A recent report by the Health Information and Quality Authority (HIQA) outlining findings from a national public engagement on health information suggested that 94% of people in Ireland think that it is important or very important for health information to be used for research ^10^. However, despite the availability of MN-CMS data and its potential to facilitate epidemiological research, the process of accessing data can be complex and there is a lack of easily accessible information outlining the procedures involved.

Therefore, the objective of this article was to provide detailed information on how a request can currently be made to access anonymised MN-CMS data on a national level for research purposes, as well as current timelines involved from initial request to data access.

## Methods

### Collaboration with clinical leads

A request to collaborate should be made to the clinical lead within each unit where data is being requested. If they agree, permission to add their name to the study protocol should be sought. It is advised that this request is made by the Principal Investigator or other senior member of the project in the first instance, as early as possible in the project timeline. Clinical leads may help to identify the appropriate person who would carry out the data extraction and anonymisation in their respective units. Additionally, it allows for an opportunity to provide further details of the project, if necessary. For example, a detailed description of the project could be presented to clinical leads/relevant persons to clarify any issues or answer any questions regarding the project, and to ensure the available data is suitable to address the proposed research question(s).

### Study protocol and data collection sheet

*Study protocol:* A detailed study protocol outlining the purpose, aims, objectives, and methodology, as well as any pertinent information surrounding data protection, governance and insurance should be drafted. The protocol should also contain information on any inclusion and exclusion criteria, for example, specific years, singleton, or multiples births and from which maternity units’ data is being requested. A study protocol will allow for ethical considerations to be clarified early on and serve as a guide for members of the research team throughout the project timeline. Furthermore, this written record of the study plan allows for monitoring of project progress and prevents unnecessary deviations from the protocol ^11^. Institutional support may be available when drafting the protocol and should be utilised where available. While currently there is no standard study protocol template for requesting anonymised MN-CMS data, Table 1 outlines an example of the key components of a study protocol.

**Table 1.** Key components of a study protocol when requesting anonymised MN-CMS data for research purposes ^11^.

1. Study title	A descriptive title (including study acronym, if applicable).
2. Funding body	Details of any funding received for the project.
3.Details of Principal Investigator and any collaborators	Names and affiliations of Principal Investigator, study collaborators or additional project members.
4. Study sites	Names of study site(s) (i.e., maternity units) from which data will be requested.
5. Background information and significance of the project	Rationale for the project - may include a short literature review.
6. Aims and objectives	Aims of the project should be clearly stated, as well as objectives of how these aims will be met.
7. Research question(s) and any hypotheses	Research question(s) should be linked to the study aims and any anticipated associations/correlations outlined.
8. Planned methodology	A detailed description of methodology including study design, study population (e.g., all singleton live births in 2023), variables being requested and all planned statistical analysis.
9. Inclusion and exclusion criteria	This may include specific years of birth, singleton, or multiples births and from which maternity units’ data is being requested.
10. Data collection and management	Details of data being requested, who will be responsible for extracting and anonymising data, how data will be transferred to project members, where data will be stored, for how long will be stored, and who will have access to the data.
11. Confidentiality	Any information relating to data protection, including a statement outlining that all data will be fully anonymised for the purpose of this project.
12. Insurance details	Details of sponsor and data controller. For example, institutional or HSE sponsorship.
13. Ethical Committee review	Outline details of each ethics committee from which approval will be sought. Permission to access data from each maternity unit will need to be applied for separately from each unit’s local Ethics Committee
14. Any amendments to the protocol	In the event of amendments to the study protocol, these should be agreed between the Sponsor and the Principal Investigator and should be recorded, submitted and approved by the relevant Ethics Committee.

Note: this is not a comprehensive list and should be adapted to specific projects. Institutional supports may be available when drafting a study protocol.

*Data collection sheet:* A data collection sheet outlining each data point/variable that is being requested for the purpose of the project must be created. This helps to ensure that the minimum amount of data is being collected for the study, and data is adequate, relevant, and limited to what is necessary for the project, reducing the risk of individuals being inadvertently identified ^12, 13^. Furthermore, it allows data manager(s) within each unit to review the requested data points and assess their availability within the MN-CMS. While currently there is no standard data collection sheet template for requesting anonymised MN-CMS data, previous data collection sheets have been created using Excel and Word software.

### National Information Governance Group

The National Information Governance Group is made up of health care professionals, scientists, informatics specialists, programme/project managers and administrators. This national group were instrumental in the initial development of the MN-CMS and continue to ensure the roll-out and on-going support of the national MN-CMS programme ^2^. Among other functions, the Information Governance group ensure compliance with relevant HSE policies and procedures as well as ensuring compliance with legislative and regulatory frameworks surrounding data protection.

If a data request is being made at a national level (i.e., from the four units that have the MN-CMS currently implemented), the protocol, data collection sheet and any other pertinent documents for the project must be reviewed and approved by the National Information Governance Group. All documents should be sent to MN-CMS Programme Manager for review at the subsequent governance group meetings, details of whom can be found here: https://www.ehealthireland.ie/ehealth-functions/acute-delivery/maternal-newborn-clinical-management-system-mn-cms/meet-the-team/meet-the-team/

### Data Protection Impact Assessment

When a decision from the National Information Governance Group is made, a Data Protection Impact Assessment (DPIA) should be completed. A DPIA helps researchers to identify risks arising from the processing of personal data and to help minimise the risks early on in the project timeline. The DPIA template can be located here: https://hseresearch.ie/data-protection-and-research/ along with a companion guide to complete the DPIA.

### Ethical Committee Review

Permission to access data from each maternity unit will need to be applied for separately from each unit’s local Ethics Committee and all local standard procedures regarding data-access must be followed. For example, ethical approval must be sought from the Clinical Research Ethics Committee of the Cork Teaching Hospitals (CREC) (for CUMH and UHK), the Research Ethics Committee of the National Maternity Hospital (for NMH) and Rotunda Hospital Research Ethics Committee (for Rotunda Hospital), prior to study commencement. Institutional support may be available when drafting ethics applications. Details on how to apply to each ethics committee are available here:

CUMH and UHK: https://www.ucc.ie/en/crec/

NMH: https://www.nmh.ie/home/research-ethics-committee.13650.html

Rotunda Hospital: https://rotunda.ie/knowledgebase/research-ethics/

### Local Information Governance Group (LIGG)

When ethical approval is granted by CREC to access data from CUMH and/or UHK, a research application form (which can be accessed from https://redcap.ucc.ie/surveys/?s=HCM7JPF8CMP3DWJH) must be submitted to LIGG. The LIGG governance group advise researchers around issues with their planned research and ethical considerations from a data governance perspective. The research application form must first be sent to the Secretary to Director of Midwifery at CUMH who will then forward the application to a LIGG representative. The LIGG meet monthly to consider all applications. Applications which are deemed appropriate for approval are then sent to the Ireland South Women and Infants Directorate, Executive Management Committee (EMC) who meet every two weeks. The Chair of the LIGG raises applications that require further discussion at the EMC meetings.

The administrative coordinator to the EMC notifies the Secretary to the Director of Midwifery of approved and/or declined applications who then informs the person named of the application form of the decision. Only requests from those affiliated with an education, healthcare or other research institution are considered.

### Data anonymisation and data transfer

According to the Data Protection Commission, data can be considered anonymised if it “does not relate to an identified or identifiable natural person or where it has been rendered anonymous in such a manner that the data subject is not or no longer identifiable”. When data is fully anonymised, it is no longer considered personal data and is therefore not subject to the same restrictions placed on the processing of personal data under the General Data Protection Regulation (GDPR). Anonymisation techniques can vary, (examples of which have been published previously ^13^) and data protection law does not prescribe any particular technique. Pseudonymisation is “the processing of personal data in such a manner that the personal data can no longer be attributed to a specific data subject without the use of additional information ^13^.

Data from the MN-CMS can be extracted and anonymised by the data manager(s) at the respective units. Therefore, project members would not have any role in the data anonymisation process. Firstly, data is extracted by data manager(s) through generation of MN-CMS reports. For example, there are approximately 30 data reports that can be automatically generated within the MN-CMS (see Table 2 for list of MN-CMS reports). Data on mothers and babies are matched using a medical record number (MRN), a unique identifier number assigned to a patient within the MN-CMS. Secondly, the data should be fully anonymised for the purpose of the project, prior to transferring it securely to project personnel. This data anonymisation process should be irreversible (i.e., not pseudonymised) and therefore no longer considered ‘personal data’ from a data protection perspective ^13^. This anonymisation process reduces the risk of data breaches, however it does not eliminate the risk and therefore precautions should be taken to avoid inadvertent data breaches. This includes precautions during the transfer of the anonymised data to project personnel.

**Table 2.** Maternal and Newborn Clinical Management System reports.

1. Active Pregnancies Report
2. Age of Women Summary Report
3. Birth Notification Report
4. Birth Weight Summary Report
5. Caesarean Section Category Report
6. Daily Discharge List Report
7. Data Quality KPI Report
8. Delivery Birth Summary Report
9. Estimated Deliveries Report
10. Estimated Deliveries Report - Future
11. Gestational Age Summary Report
12. Hypertension Report
13. Hypoxia Ischaemic Encephalopathy (HIE) Birth Report
14. Infant Breastfeeding Report
15. Maternal Deaths Report
16. Maternal Readmissions Report
17. Neonatal Discharge Report
18. Obstetric Outcome Report
19. Parity Summary Report
20. Perinatal Deaths List Reports
21. Perineal Trauma Summary Report
22. Pregnancies Opened Report
23. Pregnancy Loss Report
24. Pregnancy Outcome Report
25. Sepsis Indication Report
26. Severe Maternal Morbidity Report
27. Third Degree Tears Report
28. Total Mothers Attending Summary Report
29. Daily Delivery List Report - Long Version
30. Daily Delivery List Report - Short Version

Note: this list may vary by maternity unit.

*Data transfer:* An encrypted platform should be used when transferring the anonymised data from the maternity unit to pre-specified project personnel. One such option is FileSender, which is provided by HEAnet, Ireland's National Education and Research Network ( https://filesender2.heanet.ie/). FileSender enables users to send large, encrypted files both within and outside of academic organisations.

If requesting data from one unit only (and the researcher is affiliated with that institution), institutional-based options may be used. For example, Network Attached Storage (NAS) and cloud storage facilities such as OneDrive for Business. NAS provides a shared folder, which is made available on the institutional network, to a specified group whose members are individually authenticated by their domain logon account, while OneDrive provides a cloud storage facility within the institution to store and share work files.

### Timeline

**Figure 1.**
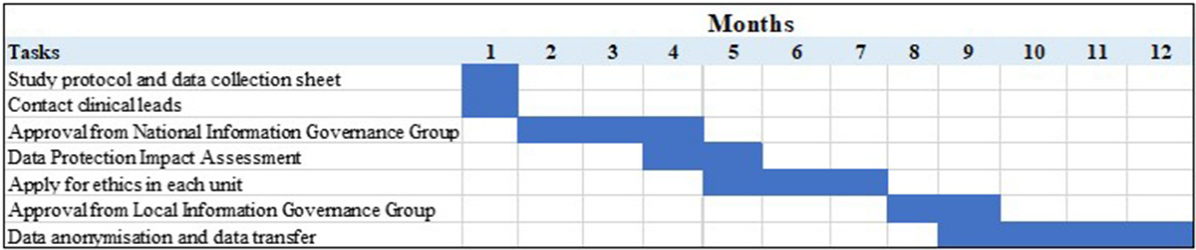
Approximate estimate of the current timeline involved between initial request and data access.

The time between initial request to data access can vary according to the amount of data being requested (i.e., number of maternity units from which data is being requested and number of variables being requested from each unit). The timeline typically ranges from approximately 6–12 months and should be factored into the project timeline. Each stage of data access can be a lengthy process, in particular if further discussion is required to clarify any information to each of the relevant bodies involved in the data request. Therefore, it is very important to provide as much detail as possible in the project protocol as well as the ethics and LIGG applications to avoid the need to clarify details, as this can result in significant delays. The Gantt chart provided in Figure 1 provides an approximate estimate of the timeline involved in each stage of data access.

## Discussion

We have provided detailed information on how a request can currently be made to access anonymised MN-CMS data for research purposes to assist researchers when applying to access data. We have also provided an estimate of current timelines involved from initial request to data access. In summary, the process can be prolonged and complex, with the timeline varying according to number of maternity units from which data is being requested and number of variables being requested.

### Strengths

There are several advantages to requesting MN-CMS data to conduct epidemiological research. First, use of secondary data for research purposes requires fewer resources than collecting primary data ^14^. In addition to this, the MN-CMS electronic health record provides valuable maternity related data on a national level which can be used to answer important research questions with an aim to make pregnancy safer and improve outcomes for mothers and babies. This is evident in other countries that use electronic health record data for research. For example, the Swedish Medical Birth Register (MBR) is an electronic health record containing information on prenatal care, delivery, neonatal care, as well as maternal sociodemographic and lifestyle factors. It was founded in 1973, and now contains data on over five million children, their mothers, and their siblings. This MBR has formed the basis of more than 1,000 research publications since its inception, the majority of which were published since the early 2000s ^15^.

### Limitations and recommendations

There are also some limitations that should be noted as well as a number of recommendations to further facilitate epidemiological research using MN-CMS data. First, while MN-CMS data are real world data and reflects outcome in practice, a reliance on existing data can be a limitation in terms of data availability, unmeasured variables, and uncertainty around data quality. However, in order to minimise issues surrounding data quality, daily data quality checks are conducted within the MN-CMS by Data Quality personnel. Any issues highlighted from these checks are then communicated with relevant healthcare personnel ^3, 16^.

Second, there is a need for an MN-CMS data dictionary to inform researchers on data availability and to support researcher analysis. This recommendation is in line with a recent report published by the Health Research Board (HRB), Ireland which highlights the need for national data dictionaries and common data formats to improve data quality, support aggregation of data and guide interpretation of findings ^9^. Furthermore, this report suggests that the many valuable health related datasets that currently exist in Ireland should be accompanied by standardised metadata in order to drive use of data resources for public benefit ^9^.

Third, the application process would likely benefit from a standard data collection sheet and protocol template. These standardised templates would need to be agreed by each unit. While we have provided details of the key components of a study protocol when requesting anonymised MN-CMS data for research purposes, this is not a comprehensive list and should be adapted to specific projects. Researchers should check if institutional supports are available when drafting the study protocol and ethics applications.

### Future directions

As the MN-CMS generates more data year-on-year, the creation of a data warehouse to manage and safely store data will likely be necessary. This should be accompanied by a national standardised data access process with transparent expected timelines and will require an adequately resourced data access system in order to drive use of MN-CMS data for research. Furthermore, the formation of a data science team to assist data management and handling of data requests would enhance the approaches to providing data for secondary use. This data science function would require increased supports from funding agencies such as Department of Health, Health Research Board and Science Foundation Ireland.

The introduction of a nominal fee for data access may also be necessary to partly cover costs associated with data processing. This fee-based model for data processing and extraction is also used in other research settings. For example, the electronic Data Research and Innovation Service (eDRIS), as part of Public Health Scotland, are the central provider of Scottish health data for use outside its primary function. The eDRIS team work in collaboration with researchers and provide support throughout the data access process. A fee applies for this service, with the cost depending on the complexity of the request and whether the request is coming from the public sector, an academic organisation, charity, commercial or private industry ^17^.

Similarly, ‘Born in Bradford’ is a birth cohort that tracks the health and wellbeing of over 13,500 children, and their parents born at Bradford Royal Infirmary between March 2007 and December 2010 ^18^. Born in Bradford does not receive funding to provide data extracts to individual projects and researchers. Therefore, a fee applies for accessing the data, and similar to the eDRIS service described above, they operate a tiered pricing structure based on the type of organisation researchers are applying from ^19^. 

Alternative models of data processing and extraction that could be considered by MN-CMS include transferring the management of data to other national agencies, as used in other research settings. For example, Hospital In-Patient Enquiry (HIPE) is the principal source of national data on discharges from acute public hospitals in Ireland. The HIPE database is managed by Healthcare Pricing Office (HPO) in the HSE, and among other tasks, are responsible for responding to requests for data from researchers ^20^. Similarly, data from Growing Up in Ireland (GUI), the national longitudinal study of children and young people, are managed by the Central Statistics Office (CSO) and researchers can apply to access GUI data through the CSO ^21^. Finally, the introduction of a single National Ethics Review Board, particularly for secondary use of data, would provide a more streamlined data access approach and would likely reduce the timeline involved between initial request and data access. 

## Conclusion

Currently, accessing MN-CMS data for research can be a complex process, including the need to interact with all the hospitals involved and the timeline from initial request to data access can range from approximately 6–12 months, depending on amount and type of data being requested. A national standardised process for managing the data is needed; this would allow a clear pathway to be developed for accessing data, along with a data dictionary, standard data collection sheet and protocol template to facilitate new data-driven discovery in the area of maternal and child health using MN-CMS data.

## Ethics and consent

Ethical approval and consent were not required.

## Data Availability

No data are associated with this article.
