# Supplementary material for: Accessing Anonymised Data from Ireland’s Maternal and Newborn Clinical Management System for Research Purposes
Source: HRB Open Res. 2024 Jun 4;7:32. [Version 1] doi: 10.12688/hrbopenres.13898.1 (PMC11883204; doi:10.12688/hrbopenres.13898.1)
Supplement: Peer review history

# Reviewer report: Reviewer response for version 1

**DOI:** 10.21956/hrbopenres.15242.r44769

**Published:** 2025-03-05

**Authors:** Antoniya Georgieva

**Affiliations:** 1 University of Oxford, Oxford, UK

This report provides details of the status quo of the accessibility for research of the maternity electronic patient (EPR) data in Ireland. I find this work really relevant and important – providing a guide to any researchers/healthcare professionals who are looking into using routinely collected EPR data.

 As the AI and ‘big data’ fields evolve, such access to anonymised data will become increasingly valuable as it can underpin improvements in healthcare provision (in the long run). But equally important, such work can allow the ability to provide audits or gather baseline data undergoing power estimates and other elements of prospective clinical trials for example.

 The report provides a critical appraisal of the current status and highlights delays and system weaknesses that could be improved. The report provides a number of useful recommendations to further facilitate

 I am not sure that this work classes as a ‘research article’ but I would think is a critical review or an appraisal of the current way to gain access or a guide to gaining access or a description of the current system. But I am not sure it is ‘research’. Importantly, it is a comprehensive guide with links to relevant materials and definitions of terms and processes.

 Overall, I would recommend the authors expand further their work with time in the following areas:

- From the Introduction: *The data are collected during routine delivery of maternity care, including but not limited to, demographics, lifestyle factors, medical history, pregnancy factors including any complications of pregnancy, delivery data such mode of delivery as well as data relating to the newborn such as birthweight and gestational age.*

It would be useful over time, to provide more details and gather information specifically around data from ultrasound images or CTG traces, or blood gas machines - i.e. any other data that may be routinely collected and stored in an electronic format. Would the authors envisage any potential use or access to those, I appreciate it would be even harder.

- The authors review existing similar initiatives, for example Born in Bradford but I was wondering if it is worth mentioning how the Irish data could be compared to data from – for example - ONS for example or the National Neonatal Research Database https://www.imperial.ac.uk/neonatal-data-analysis-unit/neonatal-data-analysis-unit/
- Example data requests may be useful to be shared as a template

Is the work clearly and accurately presented and does it cite the current literature?

Yes

If applicable, is the statistical analysis and its interpretation appropriate?

Not applicable

Are all the source data underlying the results available to ensure full reproducibility?

No source data required

Is the study design appropriate and is the work technically sound?

Yes

Are the conclusions drawn adequately supported by the results?

Yes

Are sufficient details of methods and analysis provided to allow replication by others?

Yes

Reviewer Expertise:

maternity 'big data'

I confirm that I have read this submission and believe that I have an appropriate level of expertise to confirm that it is of an acceptable scientific standard.

# Reviewer report: Reviewer response for version 1

**DOI:** 10.21956/hrbopenres.15242.r40860

**Published:** 2024-07-04

**Authors:** Rachael Wood

**Affiliations:** 1 The University of Edinburgh, Edinburgh, Scotland, UK

This paper outlines the current process of accessing data held in the maternity and neonatal electronic patient record system (MN-CMS) being implemented in hospitals across Ireland for secondary analysis/research and provides suggestions for improvement.

 The paper is well written and will be a useful resource for researchers wishing to access this new data source. I would make the following comments 

 This paper provides an interesting snapshot of a nation working to modernize its health data infrastructure to facilitate secondary use of data for research and service improvement. Important progress is clearly being made, with the introduction of a national unique patient identifier number and the incremental implementation of the national maternity and neonatal electronic patient record system MN-CMS and use of data from the system for several projects already. This paper sets out the clear, if cumbersome and time consuming, process involved in accessing the data from the MN-CMS for research.

 As someone from out with Ireland, additional context on the structure of the health service would have been helpful, for example the balance of public and private healthcare provision and how this affects data availability. I was also unsure of the status of the national information governance group, specifically whether this has an overarching remit (likely?) or governs the MN-CMS only (unlikely?).

 The authors make important suggestions for improvement. There is a clear need to improve available metadata, both on what data items are held in the MN-CMS and their associated quality. Templates to standardize study protocols and associated data requests are likely to be helpful if not too restrictive.

 The current process to obtain governance approval for data access is unlikely to lift aspiring researchers hearts. There is substantial scope to streamline the current multi-step process. Ethical risks incurred by secondary data analysis research relate primarily to privacy, but there seems to be no alignment between ethical and IG review processes.  For example, in Scotland, there are standing agreements between ethics and IG review bodies ensuring ethical review is subsumed within IG review for data driven projects if certain conditions are met. The apparent duplicate national and local IG review also appears inefficient. The requirement to complete a DPIA appears to come after national IG approval, although I would have thought that approaches to maximizing public benefit and minimizing privacy risks set out in a DPIA would usefully inform the national IG review.

 The model of data provision is of the data travelling to the researchers (through anonymization and transfer) rather than of the researcher travelling to the data (through a safe haven facility). The latter is associated with lower privacy risks.

 Overall my impression is of a very fragmented national data infrastructure. Implementation of the national unique patient identifier number is a work in progress. Even considering just data from the MN-CMS, the need for irreversible anonymization before provision to researchers will preclude identification of individuals appearing in the extracts of more than one data provider. There appears to be no ability to link MN-CMS data to other national data such as vital event, other (non-maternal/neonatal) hospital admissions, prescriptions, etc.

 Having said all this, many nations are feeling their way through the difficult business of capturing high quality data as a by-product of clinical care and using it efficiently and effectively for research to drive policy and service improvement whilst minimizing privacy risks and maintaining public trust. Ireland is to be encouraged in its journey, and this paper provides a useful insight into steps being taken relating to maternal and child health specifically.

Is the work clearly and accurately presented and does it cite the current literature?

Yes

If applicable, is the statistical analysis and its interpretation appropriate?

Not applicable

Are all the source data underlying the results available to ensure full reproducibility?

No source data required

Is the study design appropriate and is the work technically sound?

Yes

Are the conclusions drawn adequately supported by the results?

Yes

Are sufficient details of methods and analysis provided to allow replication by others?

Yes

Reviewer Expertise:

Maternal and child public health. Use of routine data for research. Epidemiology.

I confirm that I have read this submission and believe that I have an appropriate level of expertise to confirm that it is of an acceptable scientific standard.
